# Restoration of Visual Function and Cortical Connectivity After Ischemic Injury Through NeuroD1-Mediated Gene Therapy

**DOI:** 10.3389/fcell.2021.720078

**Published:** 2021-08-18

**Authors:** Yu Tang, Qiuyu Wu, Mang Gao, Esther Ryu, Zifei Pei, Samuel T. Kissinger, Yuchen Chen, Abhinav K. Rao, Zongqin Xiang, Tao Wang, Wen Li, Gong Chen, Alexander A. Chubykin

**Affiliations:** ^1^Department of Biological Sciences, Purdue Institute for Integrative Neuroscience, Purdue Autism Research Center, Purdue University, West Lafayette, IN, United States; ^2^Department of Biology, Huck Institutes of Life Sciences, Pennsylvania State University, University Park, PA, United States; ^3^Guangdong-Hong Kong-Macau Institute of CNS Regeneration, Jinan University, Guangzhou, China

**Keywords:** NeuroD1, gene therapy, functional circuit recovery, ischemic injury, visual cortex, circuit mapping, orientation selectivity, two-photon calcium imaging

## Abstract

Neural circuits underlying brain functions are vulnerable to damage, including ischemic injury, leading to neuronal loss and gliosis. Recent technology of direct conversion of endogenous astrocytes into neurons *in situ* can simultaneously replenish the neuronal population and reverse the glial scar. However, whether these newly reprogrammed neurons undergo normal development, integrate into the existing neuronal circuit, and acquire functional properties specific for this circuit is not known. We investigated the effect of NeuroD1-mediated *in vivo* direct reprogramming on visual cortical circuit integration and functional recovery in a mouse model of ischemic injury. After performing electrophysiological extracellular recordings and two-photon calcium imaging of reprogrammed cells *in vivo* and mapping the synaptic connections formed onto these cells *ex vivo*, we discovered that NeuroD1 reprogrammed neurons were integrated into the cortical microcircuit and acquired direct visual responses. Furthermore, following visual experience, the reprogrammed neurons demonstrated maturation of orientation selectivity and functional connectivity. Our results show that NeuroD1-reprogrammed neurons can successfully develop and integrate into the visual cortical circuit leading to vision recovery after ischemic injury.

## Introduction

Functional circuit impairment associated with neuronal loss is commonly seen in patients with brain injuries, such as ischemia. Though neural stem cells (NSCs) exist in the subventricular zone (SVZ) in the adult brain, they are found to differentiate mainly into astrocytes when they migrate to injured cortex (Benner et al., 2013; Faiz et al., 2015), and their neurogenesis capacity is too limited to compensate for the neuronal loss. Currently, it still remains a challenge to generate neurons in adults and functionally incorporate them into the local circuits. Several strategies have shown the capability to induce neurogenesis and lead to some behavioral recovery. One promising approach is to transplant stem cell-derived neurons or neural progenitor cells (Tornero et al., 2013; Michelsen et al., 2015; Falkner et al., 2016; Somaa et al., 2017). Yet, there are concerns about graft rejection and tumorigenicity of the transplanted cells (Erdo et al., 2003; Marei et al., 2018). Meanwhile, progress has been made in reprogramming non-neuronal cells, such as fibroblast and glial cells, into neurons directly by expressing transcription factors (Marro et al., 2011; Yang et al., 2011; Grande et al., 2013; Niu et al., 2013; Blanchard et al., 2014; Heinrich et al., 2014; Torper et al., 2015; Mattugini et al., 2019; Nolbrant et al., 2020). Brain injuries, including ischemic injury, trigger re-activation and proliferation of astrocytes around the injury site. Although the initial stage of gliosis may be beneficial to confining the injury, glial scarring in the later stage is detrimental to axonal regeneration, neural circuit rewiring, and functional recovery (Fitch and Silver, 2008; Kawano et al., 2012). One transcription factor NeuroD1, which has been demonstrated to convert human ESCs and iPSCs into neurons *in vitro* (Zhang et al., 2013), also showed great efficacy in converting astrocytes to neurons *in situ*, bypassing the pluripotent and proliferating stem cell stage (Guo et al., 2014; Chen et al., 2015; Li and Chen, 2016; Brulet et al., 2017; Chen et al., 2020), and a recent study confirmed that the converted cells originated from astrocytes using lineage tracing labeling (Xiang et al., 2021). It is possible that a small number of astrocytes that were converted originated from NSCs, but the limited number of NSCs-originated astrocytes alone in adult brains might not be able to compensate for the neuronal loss in ischemic injury. It has been shown that NeuroD1-mediated astrocyte-to-neuron conversion supported behavioral function recovery following ischemic injury in the motor cortex and the converted neurons form structural connections with thalamic neurons (Chen et al., 2020; Ge et al., 2020). Despite the demonstration of the behavior recovery, whether the newly transformed neurons integrate into the local circuits and perform appropriate functions is less clear.

Several questions remain unanswered: do the reprogrammed cells become integrated into the functional circuit in brain regions that have complex circuit structures, like cortices? Do they gain the functional properties of a typical neuron and become part of the specific circuit? Do the reprogrammed cells undergo a classical developmental path of regular neurons, or is their developmental trajectory different? Finally, how safe is this process of converting a non-neuronal cell into a neuron? Do these cells stay neurons, or do they gain other potentially aberrant cellular properties?

Answering these questions is critical for the development of new regenerative therapies for brain injuries. We have decided to answer some of these questions using the mouse primary visual cortex, which provides unique advantages as a model system. It is easily accessible for in vivo electrophysiological recordings and calcium imaging in awake mice. It is responsive to visual stimulation, providing an opportunity to characterize cortical cells’ functional properties using quantitative visual tests and various stimuli.

To examine the functional recovery of the visual cortex after ischemic injury, we directly measured neuronal activity and response selectivity in the NeuroD1-treated visual cortex in awake mice and mapped the connectivity of the individual newly reprogrammed neurons in *ex vivo* brain slices. Visual response and circuit connectivity strength were characterized longitudinally after reprogramming, revealing local circuitry remodeling and visual response recovery. Furthermore, the reprogrammed cells’ orientation selectivity improved over time as assayed by two-photon calcium imaging and extracellular recordings at different developmental stages following reprogramming. These findings suggest that NeuroD1-mediated reprogramming of astrocytes into neurons leads to neuronal regeneration and functional recovery of vision after ischemic injury.

## Experimental Procedures

### Animals

Wild type male and female C57BL/6 mice (Jackson Laboratory and Purdue University Transgenic Mouse Core Facility, postnatal day 34-90) were used for *in vivo* extracellular recording experiments. Thy1-ChR2-YFP line 18 [B6.Cg-Tg(Thy1-COP4/EYFP)18Gfng/J, JAX stock #007612] was used for *ex vivo* cortical slices preparation and whole cell patch-clamp experiments. All animals were housed in 12-h light/dark cycle with *ad libitum* access to rodent chow food and water. All experimental animal use was approved by the Purdue University Animal Care and Use Committee and followed guidance issued by the National Institutes of Health.

### Surgery, Ischemic Injury Induction, Viral Injections, and Cranial Window Installation

Mice were anesthetized during all surgical procedures with inhaled isoflurane (5% for initial induction and 1.5% for maintaining anesthesia, carrier gas was room air, SomnoSuite system). Deep anesthesia was confirmed by no response to toe/tail pinch. The skin over the skull was removed, and the skull over the cortices was exposed. The craniotomy was made first by thinning a small area of the skull about 0.5 mm diameter at the injection site with a drill. Then, a tiny gap at the center of the hole for inserting the micro-injection pipette was opened using a sterile needle. To induce focal ischemia, a total volume of 1 μl of 4 μg/μl endothelin-1 (ET-1, Sigma) was injected into V1. ET-1 was dissolved in filter-sterilized pure water to make a stock solution which was stored at −80°C and diluted to the final concentration with filter-sterilized artificial cerebral spinal fluid (ACSF) before each injection. ET-1 solution was injected at two depths, 700 μm and 300 μm below the brain surface, 500 nl per depth at 100 nl/min rate using a microinjector (NanoJect II or NanoJect III, Drummond Scientific). For sham injections, 1 μl of ACSF was injected at the same speed and depths. For mice used in extracellular recording experiments, a head post (or head plate for 2 photon imaging) was adhered to the skull at 4 mm anterior to bregma, and a gold-plated grounding pin (Parkell) was installed 1 mm anterior to bregma by inserting the sharp end through the skull into the midline space (but not in the brain tissue). Following the procedures, acrylic dental cement (Metabond, C&B) was applied to the exposed skull to create a protective hard cap and to secure the head post and the grounding pin. Ground pin installation were omitted for animals for *ex vivo* brain slice preparation and 2 photon calcium imaging. 8-10 days after ET-1 injections, two adeno-associated viruses (AAV9), one carrying FLEX-NeuroD1-mCherry and the second carrying GFAP::Cre were injected together (10:1 ratio, 1ul total volume, injected at the same depths and speed as ET-1 injection) through the same craniotomy. Coordinates used for primary visual cortices injections were (relative to lambda): 0.8 mm anterior, ±3.0 mm lateral for animals used in extracellular recordings; or 0.8 mm anterior, ±2.8 mm lateral for animals used in *ex vivo* slice recordings. For the 2 photon calcium imaging and optotagging experiments, ET-1 was injected in both hemispheres as described earlier. 8-10 days after ET-1 injection, AAV9-CAG::GFAP-Cre, AAV9-CAG::FLEX-NeuroD1-mCherry, and AAV9-CAG::FLEX-GCaMP6s (for 2 photon calcium imaging, Addgene, 100842) or AAV5-DIO-ChR2-eYFP (for optotagging, Addgene, 20298) were injected together (2:10:10 ratio) into both hemispheres at 700um and 300um below the brain surface (500 nl per depth, speed 1 nl/s). AAV9-syn-jGCaMP7s (Addgene, 104487) was injected alone into healthy mouse V1 as the healthy control for calcium imaging experiments. Carprofen (5 mg/kg) and enrofloxacin (5 mg/kg) was subcutaneously injected into mice daily after the viral injection to minimize inflammatory responses. Three days after the viral injection, a 2 mm diameter cranial window was made at the injection site using a fine dental drill. The window was then filled with sterile ACSF and covered by a 5 mm diameter glass cover. Metabond was used to seal the glass window.

### *In vivo* Extracellular Recording Preparation

Mice were habituated to the head-fixed recording setup for at least 4 days, 90 min per day, prior to recordings. Mice were head-fixed, and their bodies were loosely restrained in a tube on a platform. A monitor (21.5″ ViewSonic VX2252MH, or 25″ Alienware AW2518Hf) was positioned 16.5 cm in front of the platform showing a gray screen during habituation sessions. On recording days, small cranial windows (∼1 mm^2^) were made at the injection sites while mice were anesthetized by isoflurane inhalation. Mice were placed on the head-fixed setup after craniotomies, and a silicon probe was inserted into the cranial window. For optotagging experiments, an optical fiber (Thorlabs, 0.39NA TECS hard-clad, multimode, step-index fibers, FT200EMT) connected to a blue light laser (OEM laser, 100 mW 473 nm DPSS laser system) was positioned right above the brain surface as adjacent to the recording probe insertion site as possible. Recordings started 30 min after probe insertion to allow for recovery from anesthesia and tissue settling. Filter-sterilized ACSF was added on top of the exposed brain surface to prevent desiccation from dehydration.

### Visual Stimulation and *in vivo* Optogenetic Stimulation

All visual stimuli were generated using PsychoPy (Peirce, 2007). The full-field gray screen was used for habituation (mean luminance 73 cd/m^2^). In one stimulus recordings, sinusoidal drifting gratings (0.04 cycles per degree, drifting at 2 Hz, oriented 30 degrees to the vertical direction) were presented for 20 trials. In each trial, the stimulus was presented for 0.2 s, preceded by 0.5 s gray screen, followed by 5-6 s gray screen inter-trial interval. For direction tuning recordings, sinusoidal drifting gratings (0.04 cycles per degree, drifting at 1 Hz, oriented 0, 30, 60, 90, 120, and 150 degrees) were pseudo-randomly presented for 60 trials. Within each trial, the stimulus was presented for 1 s, preceded by 0.5 s gray screen, followed by 5-6 s inter-trial interval. For optogenetics experiments, light stimulation was applied after all visual stimulation experiments to identify cells that co-express NeuroD1 and ChR2. 500 ms light pulses (5-10 mW measured at the fiber tip) were applied for 20 trials.

### Extracellular Recording Data Acquisition and Analysis

A 64-channel silicon probes (Shobe et al., 2015) were used for all recordings. Raw data were digitized at 30 kHz and acquired through an OpenEphys acquisition board (Siegle et al., 2017). Local field potentials were obtained by band-pass filtering the raw data between 0 and 300 Hz with an additional 60-Hz notch filter to attenuate electrical noise. The channels within the depth range of layer 4 (300 to 500 μm below the brain surface) that showed the first strongest negative response to visual stimulation were used for visually evoked potential (VEP) analysis. The most negative value within the visual stimulation time window was used as the VEP amplitude. Time-frequency analysis of LFP was performed by using a series of complex wavelets to extract power and phase at each sample point. Band powers were calculated by averaging powers within 500 ms after the visual stimulation onset.

Spikes were clustered into units using Kilosort (Pachitariu et al., 2016a). Units were then manually inspected in Phy (Rossant et al., 2016) template graphical user interface (GUI) to remove units that have noise-like waveforms (artifact-like or have no clear refractory period). Single units were classified as regular-spiking (RS), fast-spiking (FS), and unclassified (UN) units, based on their averaged template waveforms. Units that have averaged template waveform with trough-to-peak duration less than 0.45 ms and spike width less than 1 ms were classified as putative FS units. Units that had template waveform with trough-to-peak duration more than 0.45 ms and spike width more than 1 ms were classified as RS units. Units that did not satisfy either criterion were classified as UN units. Spike width was calculated by inverse peak frequency of the spike spectrum (Stark et al., 2013). Peri-stimulus time histograms (PSTHs) of single unit activities were computed using 10 ms bins and smoothed with a Gaussian Kernel (width = 100 ms). Z-scores of single unit firing rate (FR) were calculated by normalizing FR to the mean FR across the duration of each trial [z = (FR-mean FR)/standard deviation of FR]. Mean FR within the visual stimulation time window was used as the response to each direction. FR at each orientation was obtained by averaging the FR at the same orientation of two directions. Tuning curves for each group were obtained by fitting unit averaged FR at 6 orientation to Gaussian functions or by interpolating a cubic function. One minus direction circular variance (1-DCV) was calculated using $\left|\frac{\sum_{k}R\left(\theta_{k}\right)\text{exp}\left(i\theta_{k}\right)}{\sum_{k}R\left(\theta_{k}\right)}\right|$, and one minus orientation circular variance (1-CV) was calculated using $\left|\frac{\sum_{k}R\left(\theta_{k}\right)\text{exp}\left(2i\theta_{k}\right)}{\sum_{k}R\left(\theta_{k}\right)}\right|$, where θ_*k*_ was the direction *k* (0-2π) or orientation *k* (0-π) in radians, and *R*(θ_*k*_) was the mean firing rate within the stimulus time window (Mazurek et al., 2014).

### Two Photon Calcium Imaging Data Acquisition and Analysis

Two photon calcium imaging was performed using a Nikon A1R-MP-HD multiphoton microscope with a 16× 0.8 NA water-dipping objective lens (Nikon LWD 16X W) and dual Coherent IR lasers (Chameleon Discovery). Awake mice were head fixed under the microscope, and the image was acquired through the cranial window. The imaged area was around 350 μm × 350 μm, and the images were acquired at 512 × 512 pixels. The GCaMP6s signals were excited at 890 nm, and were acquired by a 4-channel episcopic GaAsP Non-descanned Detector with a resonant mode at 30 fps. During the time series image acquisition, sinusoidal drifting gratings of 12 directions (5 s for each direction, interleaved by 10 s gray screens at the same luminance as the stimuli) were presented for 12 trials. After the calcium imaging, the NeuroD1-mCherry signal of the same image plane was acquired with galvanometer mode.

Time series calcium data were processed using Suite2P (Pachitariu et al., 2016b) with non-rigid mode allowing for 10% X-Y axial movements. ROIs were first extracted automatically and then were manually inspected to remove non-cell-like ROIs. To minimize signal contamination by surrounding cells and neuropil, 0.7 times of the spatially averaged neuropil’s signal was subtracted from the ROI (Chen et al., 2013). The slow fluctuation was removed by subtracting means of 100 s running windows from the signal. The relative calcium signal change was calculated as the ratio of fluorescence change to 2 s baseline fluorescence (Δf/f_0_). The response latency was measured as the duration between the stimulus onset to the time point when Δf/f_0_ was higher than 1.675 times of baseline standard deviation. To take astrocyte visual response into account, two response time windows were defined. One is 5 s duration from the stimulus onset (for neurons), and the other is 5 s duration from 3 s post-stimulus onset (for astrocytes). Temporally averaged Δf/f_0_ within these two time windows that were significantly higher than baseline (Wilcoxon signed-rank test, *p* < 0.05) and has a response latency that was less than 8 s were considered as visual responses. Cells that respond to at least one direction were used for further analyses. Visually evoked response latency of a cell was defined as the minimal latency among latencies of all directions. The circular orientation-selective index (1-CV) and the circular direction-selective index (1-DCV) were calculated as described earlier.

### Acute Brain Slices Preparation

Mice were anesthetized with an intraperitoneal (IP) injection of a cocktail of ketamine (100 mg/kg body weight) and xylazine (16 mg/kg body weight) diluted in sterile saline. Deep anesthesia was confirmed with no reflex to toe/tail pinch. For animals that were at the age of 55 days or younger, trans-cardiac perfusion was conducted using ice-cold High Sucrose Dissection Buffer (HSDB) containing (in mM) 75 sucrose, 10 glucose, 87 NaCl, 2.5 KCl, 1.25 NaH_2_PO_4_, 25 NaHCO_3_, 0.5 CaCl_2_, 7 MgCl_2_, and 1.3 ascorbic acid. Following perfusion, the brain was quickly dissected out of the skull, and the visual cortex was cut on a vibratome (VT1000, Leica) into slices at 300 μm thickness in ice-cold HSDB. Brain slices were then carefully transferred into normal Artificial Cerebral Spinal Fluid (ACSF) containing (in mM) 124 NaCl, 3.5 KCl, 1 CaCl2, 0.8 MgCl2, 1.23 NaH2PO4, 26 NaHCO3, and 10 glucose. The slices were first incubated at 32°C in ACSF for 30 min then at room temperature (around 25°C) for 1 to 6 h before recording. For animals that were older than 55 days, trans-cardiac perfusion was conducted using ice-cold N-methyl-D-glucamine (NMDG) ACSF containing (in mM) 92 mM NMDG, 2.5 mM KCl, 1.25 mM NaH_2_PO_4_, 30 mM NaHCO_3_, 20 mM HEPES, 25 mM glucose, 2 mM thiourea, 5 mM Na-ascorbate, 3 mM Na-pyruvate, 0.5 mM CaCl_2_ and 10 mM MgCl_2_. Dissection and slicing were conducted in the same manner as for young animals but in ice-cold NMDG ACSF. Brain slices were then recovered in NMDG ACSF at 32°C for 4 to 7 min depending on the animal age, then in HEPES ACSF containing (in mM) 92 mM NaCl, 2.5 mM KCl, 1.25 mM NaH_2_PO_4_, 30 mM NaHCO_3_, 20 mM HEPES, 25 mM glucose, 2 mM thiourea, 5 mM Na-ascorbate, 3 mM Na-pyruvate, 2 mM CaCl_2_ and 2 mM MgCl_2_ at room temperature for at least 2h before recording. All physiological solutions were continuously aerated with carbogen gas (95% O_2_ 5% CO_2_) to maintain pH (7.3-7.4) and oxygen saturation. Brain slices were kept alive for up to 7 h after cutting and each recorded slice was used for up to 1.5 h.

### Whole-Cell Patch Clamp Recordings

Patch-clamp recordings were conducted using a commercial slice physiology rig (SliceScope Pro 1000, Scientifica). Patch pipettes were pulled using a standard Flaming-Brown type puller (Sutter Instruments P97) from filamented borosilicate glass capillaries (BF150-86-10, Sutter Instruments). The pipette internal solution contained (in mM) 20 KCl, 100 K-gluconate, 10 HEPES, 4 MgATP, 0.3 Na2GTP, and 7 phosphocreatine, with pH adjusted to 7.4 and osmolarity adjusted to 300 mOsm. In some experiments, a small amount of 4% w/v Alexa Fluor^TM^ 647 Hydrazide (A20502, Thermo Fisher Scientific) dissolved in internal solution was back-loaded to the glass pipette through capillary force before loading the regular internal solution to label the patched cell. Pipette impedance was in the range of 3.5-7.9 MΩ when filled with internal solution and submerged in ACSF. Brain slices were placed in a recording chamber continuously perfused with oxygenated ACSF and heated to 30-32°C. Cells were visualized with infrared illumination through differential interface contrast (DIC) optics and recorded with a charge-coupled device (CCD) camera. Signals were amplified using a Multiclamp 700B amplifier (Molecular Devices) and digitized using Digidata 1550A (Molecular Devices) at 20 kHz and low-pass filtered at 10 kHz. Recorded data were analyzed using custom-written Python scripts (detailed statistical tests see “Experimental design and statistical analysis”). For experiments during where cells were filled with fluorescent dyes, the slices were fixed with 4% paraformaldehyde (PFA) for 30 min-1 h and mounted onto glass slides for imaging.

### Channelrhodopsin-Assisted Circuit Mapping (CRACM)

We used Thy1-ChR2-YFP line 18 (B6.Cg-Tg(Thy1-COP4/EYFP)18Gfng/J, JAX stock #007612) which expressed Channelrhodopsin-2 (ChR2) sparsely in layer 5 pyramidal cells in the cortex (Arenkiel et al., 2007). To control for intrinsic synaptic strength difference in different projections, only layer 4 neurons were patched, only L5 to L4 projections were compared. L4 cells were identified by the morphology and relative location in the brain slice (mid-point from pia to white matter). Light stimulation was generated with an LED light source (High-Power LED Collimator Source, 470 nm, 50W, Mightex) and delivered through a patterned illuminator (Polygon 400, Mightex) (Avants et al., 2015). A 10 by 10 grid covering a 670 μm by 670 μm square area was superimposed on the primary visual cortical slice, which spans the top border of L2/3 to the lower border of L5 under 10x objective. Each pixel was stimulated for 10 ms, following a pseudo-random sequence with 2 s inter-stimulus interval. Cells were held at -70 mV in voltage-clamp mode during CRACM recordings. The LED and patterned illuminator were controlled by the manufacturer’s software, and stimulation and recording were synchronized by the Digidata. CRACM heatmaps were plotted from light-induced EPSC amplitudes at each pixel.

### Histology and Immunohistochemistry (IHC)

Mice were anesthetized with 100 mg/kg ketamine and 16 mg/kg xylazine through IP injection before trans-cardiac perfusion. Deep anesthesia was confirmed with no reflex to toe/tail pinch. The thorax and abdomen were opened. A needle was inserted into the left ventricle of the heart, and a small incision was made in the right atrium. Mice were first perfused with 1x phosphate-buffered saline (PBS, 15 to 20 ml) until the liver cleared, then with 4% paraformaldehyde (PFA, 10 to 15 ml) for fixation. Mouse brains were post-fixed in 4% PFA for an additional 12-36hr before histology. Fixed brain tissue was sliced using a vibrating microtome (1000 Plus, TPI Vibratome) at 50 μm thickness. When IHC staining was unnecessary, slides were made directly by mounting the slices with anti-fade mounting medium containing 0.2% n-propyl gallate. When IHC is necessary, the 50 μm slices were stained free-floating in 24-well tissue culture plates. They were first blocked and membrane permeabilized in 5% bovine serum albumin (BSA) and 0.1%-2% Triton X 100 (TX 100, Sigma) in PBS at room temperature for 0.5-1 h. Then, the slices were incubated with the primary antibody in 0.1% TX 100 for 36 to 48h at 4°C followed by the secondary antibody for 1 to 2h at room temperature. Slices are washed in PBS in between antibody incubations. Slices were counterstained with DAPI when necessary. For Ctip2 and Satb2 staining, slices were treated in 80°C sodium citritate buffer for 20 min before blocking. The slices were mounted using the same method described above. Antibodies used are: Anti-Glial Fibrillary Acidic Protein Antibody (AB5541, Millipore Sigma), Anti-NeuN Antibody (ABN78, Millipore Sigma), Anti-Satb2 Antibody (ab51502, abcam), Anti-GABA Antibody (A2052, Sigma), Anti-Cux1 Antibody (11733-1-AP, proteintech), Anti-Ctip2 Antibody (ab18465, Abcam), Anti-Tbr1 (AB10554, Millipore Sigma), Alexa Fluor^®^ 488 AffiniPure Donkey Anti-Rabbit IgG (H+L) (Code: 711-545-152, Jackson ImmunoResearch), Alexa Fluor^®^ 647 AffiniPure Donkey Anti-Rat IgG (H+L) (Code: 712-605-150, Jackson ImmunoResearch), and Alexa Fluor^®^ 647 AffiniPure Goat Anti-Chicken IgY (IgG) (H+L) (Code: 103-605-155, Jackson ImmunoResearch). Brain slices were imaged under a confocal microscope (Zeiss LSM710). Neurite tracing and reconstruction was conducted using Fiji/ImageJ. For quantification of marker positive reprogrammed cells, ROIs were identified on the mCherry channel, and the intensities of the markers were measured within ROI. Normalized intensity above threshold (1.5 times of median intensity of each slice) is considered as positive marker cell.

### Experimental Design and Statistical Analysis

Experimental groups and controls are described in detail with the results. Data were analyzed using custom-written scripts in Python. Data normality was tested using the Shapiro-Wilk normality test and statistically tested using the Scipy, Statsmodels, or Pingouin (Vallat, 2018) statistical packages. For normally distributed data, a Student’s *t*-test was used for pair-wise comparisons, or ANOVA was used for comparison among multiple groups. For non-normally distributed data, non-parametric tests were used. Mann-Whitney *U*-test was used for comparing two distributions with similar shape; Kruskal-Wallis *H*-test was used for comparing multiple distributions with similar shape, and the Kolmogorov-Smirnov test was used for comparing two distributions with different shapes. Group distributions of VEP amplitudes, LFP frequency band powers, unit firing rate z-scores, EPSCs amplitude were compared using non-parametric Mann-Whitney *U*-test with effect size reported. Unit counts were compared using Kruskal-Wallis *H*-test. 1-DCV and 1-CV cumulative distributions were compared using the two-sample Kolmogorov-Smirnov test. When comparing multiple normal distributions or converted normal distributions of EPSCs amplitude, one-way or two-way ANOVA was conducted, followed by Tukey’s *post hoc* multiple comparisons test. For averaged EPSCs data, either Box-Cox or log transformation was applied to get normal distributions (Young 3 weeks: Box-Cox, lambda = −0.245; Young 6 weeks: Box-Cox, lambda = −0.836; Old 6 weeks: log_10_ transformation). See the results section for the specific test used in each case and the test statistic values.

## Results

### NeuroD1 Efficiently Converted Astrocytes Into Neurons That Acquired Cortical Neuron Identity in the Visual Cortex

**FIGURE 1:**
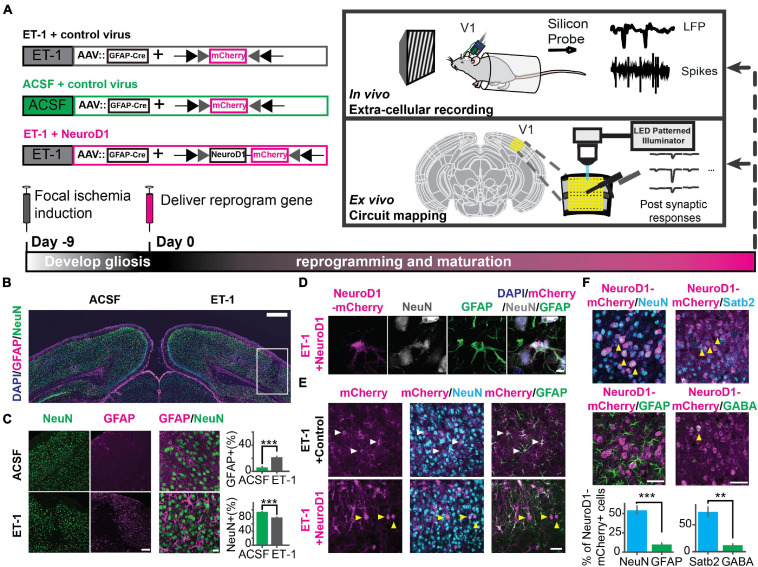
Focal ischemic injury model and *in vivo* direct reprogramming in the primary visual cortex. **(A)** The schedule to induce focal ischemia by endothelin-1 (ET-1) injection and to reprogram astrocytes into neurons by injection of AAV-GFAP::Cre with AAV-FLEX-NeuroD1-mCherry (or with AAV-FLEX-mCherry as control). Following reprogramming, visual responses and local circuit connectivity of the primary visual cortex (V1) were assessed by *in vivo* extracellular recordings and *ex vivo* Channelrhodopsin-Assisted Circuit Mapping (CRACM). **(B)** Gliosis (GFAP, magenta) and neuronal loss (NeuN, green) at 9 days after 4 ug/ul ET-1 injection in the visual cortex. The box indicates injury site with gliosis and neuronal loss. Scale: 500 μm. **(C)** Localized neuronal loss and gliosis at 9 days after 4ug/ul ET-1 injection. Scale_left_: 50 μm. Scale_right_: 20 μm. Right: Quantification of the percentages of GFAP-positive cells (top) and NeuN-positive cells (bottom). N_ACSF_ = N_ET–1_ = 3 mice, 9 slices. *p* = 4.12 × 10^–4^, Mann-Whitney U test. **(D)** An example of NeuroD1-mCherry-positive cell undergoing a transition stage at 10 days after NeuroD1 delivery, expressing both GFAP and NeuN. Scale: 5 μm. **(E)** mCherry positive cells co-stained with NeuN and GFAP at 10 days after viral injection. White arrows pointing to GFAP positive and NeuN negative cells. Yellow arrows pointing to GFAP negative and NeuN positive cells. Scale: 50 μm. **(F)** NeuroD1-positive cells stained with NeuN, GFAP, Satb2, and GABA at 3 weeks after NeuroD1 delivery. Scale: 50 μm. Bottom: Quantification of the ratios of marker positive cells. N_NeuN/GFA__P_ = 3 mice, 9 slices. *p* = 9.01 × 10^–4^, Mann-Whitney *U*-test. N_Satb2/GABA_ = 2 mice, 6 slices. *p* = 5.08 × 10^–3^, Mann-Whitney *U*-test. **p < 0.01 and ***p < 0.001. Data are represented as mean ± SEM. See also Supplementary Figures 1, 2.

**FIGURE 2:**
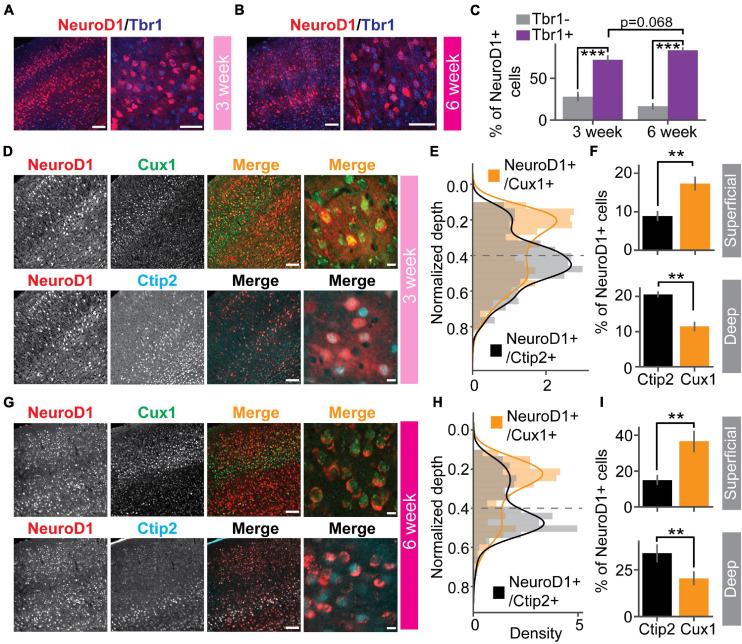
Reprogrammed cells acquire superficial and deep cortical layer identities. **(A)** NeuroD1 positive cells stained with cortical neuron marker Tbr1 at 3 weeks after the viral injection. Scale_left_: 100 μm, Scale_right_: 50 μm. **(B)** Same as **(A)** but at 6 weeks after the viral injection. Scale_left_: 100 μm, Scale_right_: 50 μm. **(C)** Quantification of Tbr1 positive cells out of NeuroD1 positive cells. Tbr1- vs. Tbr1+: N_3 week_ = 2 mice, 12 slices, *p* = 1.23 × 10^–4^; N_6 week_ = 4 mice, 23 slices, *p* = 1.51 × 10^–8^. 3 week vs. 6 week: *p* = 0.068. Mann-Whitney *U*-test with Bonferroni correction. **(D)** A representative slice showed that NeuroD1 positive cells co-stained with superficial layer marker Cux1 (top row), deep-layer marker Ctip2 (bottom row) at 3 weeks after the viral injection. The NeuroD1 image at the top and bottom row is the same. Scale_left_: 100 μm, Scale_right_: 10 μm. **(E)** Probability density of counts of NeuroD1+/Cux1+ cells and NeuroD1+/Ctip2+ cells across normalized cortical depths at 3 weeks after the viral injection. 0 indicates the brain surface. The dashed line indicates superficial and deep layer separation. **(F)** Percentages of Cux1+/NeuroD1+ and Ctip2+/NeuroD1+ cells in the superficial layers (top) and the deep layers (bottom) at 3 weeks after the viral injection. N_superficial_ = 2 mice, 4 slices, *p* = 3.06 × 10^–3^. N_dee__p_ = 2 mice, 4 slices, *p* = 2.54 × 10^–3^. Mann-Whitney *U*-test with Bonferroni correction. **(G)** Same as **(D)** but at 6 weeks after the viral injection. **(H)** Same as **(E)** but at 6 weeks after the viral injection. **(I)** Same as **(F)** but at 6 weeks after the viral injection. N_superficial_ = 3 mice, 7 slices, *p* = 3.06 × 10^–3^. N_dee__p_ = 3 mice, 7 slices, *p* = 2.33 × 10^–3^. Mann-Whitney *U*-test with Bonferroni correction. **p < 0.05 and ***p < 0.001. Data are represented as mean ± SEM.

To demonstrate the effects of *in vivo* direct reprogramming on visual function following cortical ischemic injury, we assessed visual responses using *in vivo* extracellular recordings in awake mice. To measure how newly converted neurons integrated into the local cortical circuits, we used *ex vivo* channelrhodopsin-assisted circuit mapping (CRACM) in acute brain slices (Figure 1A). At nine days after endothelin-1 (ET-1) injection, robust gliosis and neuronal loss were confirmed by astrocyte marker GFAP and neuronal marker NeuN staining, which showed a significant increase in astrocyte/neuron ratio (Figures 1B,C). The induced glial scar did not resolve if no treatment was applied (Supplementary Figure 1A, middle). After the glial scar was formed, we delivered the Cre-dependent reprogramming gene NeuroD1 (CAG::FLEX-NeuroD1-mCherry) along with the Cre-recombinase gene under the GFAP promoter (GFAP::Cre) targeting astrocytes using adeno-associated virus (AAV9). The astrocytes underwent a transition to neurons, where they temporarily expressed both GFAP and NeuN (Figure 1D and Supplementary Figure 1B, middle). The fully reprogrammed neurons only expressed NeuN, but not GFAP, which was detected as early as ten days after the viral injection (Figure 1E, yellow arrows). 3 weeks after the viral injection, more than 50% of NeuroD1-mCherry positive (NeuroD1-mCherry+) cells expressed only NeuN but not GFAP (Figure 1F, left). The exogenous NeuroD1 expression was significantly higher compared to the endogenous expression (Supplementary Figure 2). The majority of NeuroD1-mCherry+ expressed excitatory neuronal marker Satb2, and a small percentage expressed GABAergic cell marker (Figure 1F, right). Furthermore, we tested whether the reprogrammed neurons acquired cortical neuron identity and whether they formed layer structure by immunostaining a cortical neuronal marker Tbr1, a superficial layer marker Cux1, and a deep layer marker Ctip2. At both 3 and 6 weeks after the viral injection, more than 50% of NeuroD1-mCherry+ cells expressed Tbr1 (Figures 2A–C), indicating their cortical neuron identity. The NeuroD1-mCherry+ cells within the superficial and deep layers were immunopositive for Cux1 and Ctip2, respectively. The percentage of Cux1+/NeuroD1+ cells was higher in the superficial layers, lower in the deep layers, compared to Ctip2+/NeuroD1+ cells (Figures 2D–I). These results demonstrate that NeuroD1 efficiently converts astrocytes to neurons, which acquire cortical neuron identities and form cortical layer structure, allowing for functional circuit integration.

### *In vivo* Direct Reprogramming Recovered Visually Evoked Potentials (VEPs) and Single-Unit Responses

**FIGURE 3:**
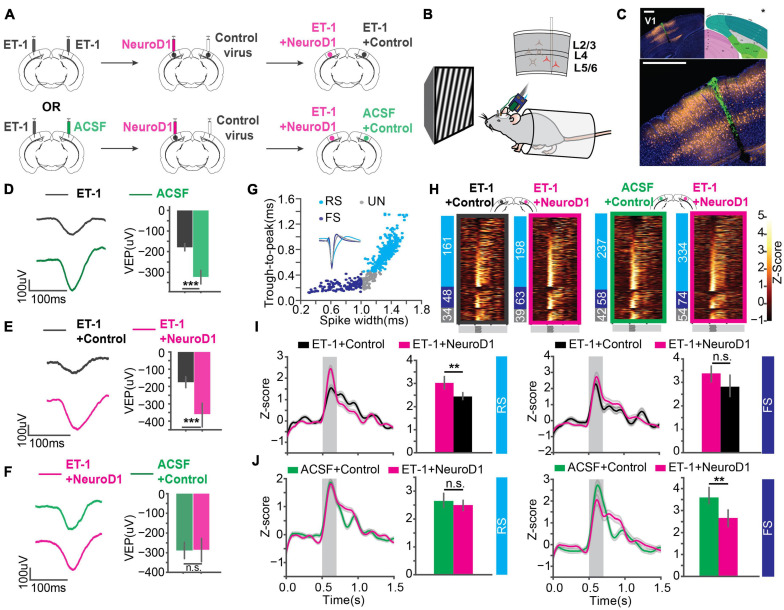
*In vivo* direct reprogramming recovers visually evoked potentials (VEPs) and single unit visual responses. **(A)** The injection scheme for *in vivo* experiments. **(B)** The *in vivo* awake extracellular recording setup. **(C)** Histology showing the probe track (green) within the reprogramming site. NeuroD1: orange, DAPI: blue. *The brain atlas is adapted from ©Allen Institute for Brain Science. Allen Adult Mouse Atlas. Available from: atlas.brain-map.org. Scale: 500 μm (top and botom). **(D)** Averaged VEPs of ET-1 and ACSF hemispheres. Quantification of VEP amplitudes on the right in each panel. N_1_ = N_2_ = 27 recording sites, 9 mice, *p* = 3.07 × 10^–4^, Mann-Whitney *U*-test. **(E)** Same as **(D)** but for ET-1+Control and ET-1+NeuroD1 hemispheres. N_1_ = N_2_ = 33 recording sites, 11 mice, *p* = 1.30 × 10^–5^, Mann-Whitney *U*-test. **(F)** Same as **(D)** but for ACSF+Control and ET-1+NeuroD1 hemispheres. N_1_ = N_2_ = 30 recording sites, 10 mice, *p* = 0.559, Mann-Whitney *U*-test. **(G)** Units classified into regular-spiking (RS), fast-spiking (FS), and unclassified (UN) units. Scatter plot showing trough-to-peak latency and waveform width of the units. The averaged template waveforms shown in the inset. **(H)** Firing rate z-scores of all units in heatmaps for “ET-1+Control vs. ET-1+NeuroD1” and “ACSF+Control vs. ET-1+NeuroD1” groups. The numbers of RS, FS, and UN units are shown on the left for each heatmap. **(I)** Left: Firing rate z-scores of RS units in the “ET-1+Control vs. ET-1+NeuroD1” group. The shaded area: visual stimulation. Bar graph showing peak z-scores within the visual stimulation window. N_ET–1__+__Control_ = 161 units, 11 mice, N_ET–1__+__NeuroD1_ = 198 units, 11 mice, *p* = 1.56 × 10^–3^, Mann-Whitney *U*-test. Right: Same as the left but for FS units. N_ET–1__+__Control_ = 48 units, 11 mice, N_ET–1__+__NeuroD1_ = 63 units, 11 mice, *p* = 0.084, Mann-Whitney *U*-test. **(J)** Left: Firing rate z-scores of RS units in the “ACSF+Control vs. ET-1+NeuroD1” group. The shaded area: visual stimulation. Bar graph showing peak z-scores within the visual stimulation window. N_ACSF__+__Control_ = 237 units, 10 mice, N_ET–1__+__NeuroD1_ = 334 units, 10 mice, *p* = 0.238, Mann-Whitney *U*-test. Right: Same as the left but for FS units. N_ACSF__+__Control_ = 58 units, 10 mice, N_ET–1__+__NeuroD1_ = 74 units, 10 mice, *p* = 0.0058, Mann-Whitney *U*-test. *p < 0.05, **p < 0.01, and ***p < 0.001, n.s., not significant. Data are represented as mean ± SEM.

To assess functional recovery of V1 after reprogramming, we recorded visually evoked potentials (VEPs) and single-unit spikes in awake head-fixed mice using extracellular recording technique. Mice were separated into two groups (Figure 3A). In one group, ET-1 ischemia was induced in both hemispheres, followed by reprogramming (FLEX-NeuroD1-mCherry) in one hemisphere and the other hemisphere injected with a control virus (FLEX-mCherry) (“ET-1+NeuroD1 vs. ET-1+Control”). In the other group, ischemia was induced in only one hemisphere followed by reprogramming (FLEX-NeuroD1-mCherry), and the other hemisphere was sham-injected (ACSF) and treated with the control virus (“ET-1+NeuroD1 vs. ACSF+Control”). Mice were habituated to the head-fixation setup prior to experiments. 3 weeks after viral injections, visual response to sinusoidal drifting gratings was recorded with a silicon probe (recording site validated with histology, Figures 3B,C). VEPs were compared between the two hemispheres within the same mouse to control for individual variability across animals. To validate visual function impairment by ET-1 induced ischemia, we added a group of mice, which were given only ET-1/ACSF injection. VEP amplitudes were significantly smaller in the ET-1 injected hemispheres compared to the ACSF injected hemispheres (Figure 3D). Next, we tested “ET-1+NeuroD1 vs. ET-1+Control” mice. VEP amplitudes were significantly larger in the ET-1+NeuroD1 hemispheres than the ET-1+Control hemispheres (Figure 3E). In contrast, for the “ET-1+NeuroD1 vs. ACSF+Control” group, VEP amplitudes were not significantly different between the two hemispheres (Figure 3F). In addition to the synchronized population activity, we also examined the single unit visual responses. Considering the heterogeneity of cortical neurons, we split the units into putative regular-spiking (RS) and fast-spiking (FS) units, based on trough-to-peak latencies and waveform latencies of their averaged template waveforms (Figure 3G). Based on both intracellular and extracellular studies, excitatory pyramidal neurons show regular-spiking waveforms, while inhibitory interneurons show fast-spiking waveforms (Connors and Gutnick, 1990; Henze et al., 2000; Trainito et al., 2019). To account for different baseline activity across units, we calculated the z-scores of firing rate over time for each unit. Z-scores of the visually evoked responses of all recorded units are shown in heatmaps (Figure 3H). In the “ET-1+NeuroD1 vs. ET-1+Control” group, RS units in the ET-1+NeuroD1 hemispheres showed significantly higher peak firing rate z-scores compared to the ET-1+Control hemispheres (Figure 3I, left). In the same group, FS units in the ET-1+NeuroD1 hemispheres showed similar peak firing rate z-scores compared to the ET-1+Control hemispheres (Figure 3I, right). In the “ET-1+NeuroD1 vs. ACSF+Control” group, RS units in the ET-1+NeuroD1 hemispheres showed comparable peak firing rate z-scores to the ACSF+Control hemispheres (Figure 3J, left). Interestingly, FS units in the ET-1+NeuroD1 hemispheres showed lower peak firing rate z-scores than the ACSF+Control hemispheres (Figure 3J, right). These results demonstrate that ET-1 induced ischemia significantly impairs visual response in V1, and *in vivo* direct reprogramming restores the visual responses, to a comparable level as in the sham condition. The single-unit activity suggests that there may be a differential recovery of visual responses in RS and FS cells. At 3 weeks post-infection, RS cells have regained normal levels of visual responsiveness, while FS cells have not.

### Reprogrammed Neurons Were Integrated Into the Local Circuit and Hyper-Connected at an Early Stage

**FIGURE 4:**
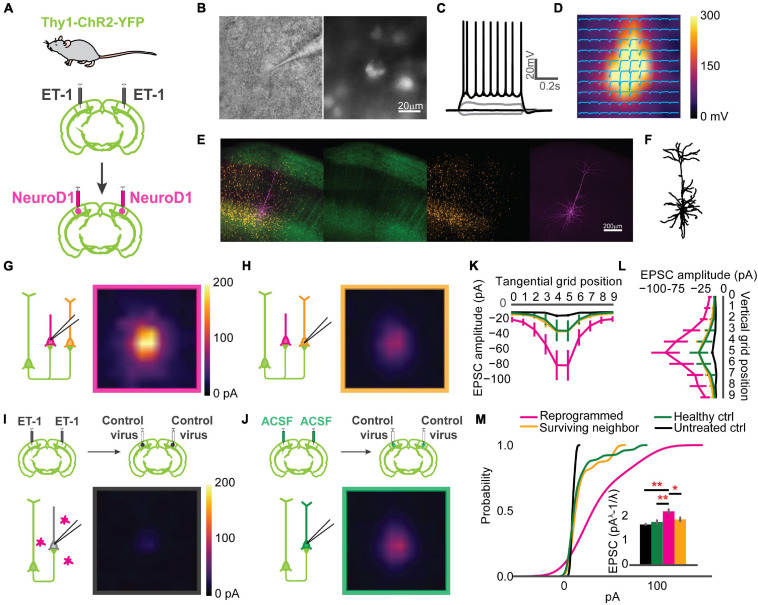
Reprogrammed neurons are integrated into the local circuit and hyper-connected at an early stage. **(A)** Thy1-ChR2-YFP mice used for Channelrhodopsin-Assisted Circuit Mapping (CRACM) experiments. **(B)** An example whole-cell patch in Differential Interface Contrast (DIC) image (left) and epifluorescence image (right), showing a mCherry positive cell. **(C)** The cell in panel **(B)** showing evoked action potentials after step-current injection. **(D)** EPSC amplitudes of the cell in panel **(B)** shown in a CRACM heatmap overlaid with EPSC traces at each stimulation point. **(E)** Confocal images of showing a layer 5 neurons expressing ChR2-YFP (green) and reprogrammed cells expressing mCherry (orange). The patched cell filled with dye (magenta). Scale: 200 μm. **(F)** Morphology of the cell processes from images in panel **(E)**. **(G)** Averaged CRACM heat map of mCherry positive reprogrammed cells (*N* = 21 cells from 6 mice). **(H)** Same as **(G)** but for mCherry negative survived neighbors (*N* = 16 cells from 6 mice). **(I)** Averaged CRACM heat maps of untreated ischemia control (**I**, *N* = 19 cells from 4 mice). **(J)** Same as **(I)** but for healthy control (*N* = 26 cells from 5 mice). **(K)** Averaged maximal EPSC ± SEM by grid position in the tangential direction (parallel to the brain surface). **(L)** Averaged maximal EPSC ± SEM by grid position in the vertical direction (perpendicular to the brain surface). **(M)** Cumulative density of EPSCs from each group. The inset bar graph showing Box-Cox-transformed EPSCs amplitude comparison among groups. Reprogrammed vs. Untreated: *p* = 0.001; Reprogrammed vs. Healthy: *p* = 0.001; Reprogrammed vs. Surviving: *p* = 0.0178, One-way ANOVA followed by Tukey *post hoc* tests. *p < 0.01 and **p < 0.05, from Tukey’s HSD. Data are represented as mean ± SEM.

Paired recordings are used to measure the strength of the connection between neighboring neurons. However, the probability of finding a connected pair decreases drastically with increasing distance, making it unsuitable for any specific interlaminar or long-range synaptic connections. Channelrhodopsin-assisted circuit mapping (CRACM) can measure the strength of the specific genetically labeled long-range and interlaminar connections (Petreanu et al., 2007; Yamawaki and Shepherd, 2015). To directly measure the circuit connectivity of newly reprogrammed neurons, we used CRACM in *ex vivo* acute slices. We used heterozygous Thy1-ChR2-YFP mice that have a sparse distribution of ChR2-eYFP positive cells among the layer 5 pyramidal cells in V1 (Arenkiel et al., 2007). Focal ischemia induction and reprogramming viral injection procedures were the same as for mice used for *in vivo* recordings (Figure 4A). Age-matched mice were randomly assigned to three groups: Healthy control (ACSF+Control), Untreated control (ET-1+Control), and reprogrammed (ET-1+NeuroD1). For each mouse, both hemispheres received the same treatment. We further divided neurons in the reprogrammed group into mCherry-positive reprogrammed cells and mCherry-negative surviving neighbors. We then performed whole-cell patch-clamp recordings using differential interference contrast (DIC) microscopy and fluorescent image-guided targeting (Figure 4B). Basic electrophysiological properties were characterized by recording the membrane potential changes following a series of step currents for each cell (Figure 4C). Then, CRACM maps were collected in the presence of TTX and 4-AP to isolate mono-synaptic connections (Figure 4D). For some mCherry-positive reprogrammed cells, a fluorescent dye was included in the patching pipette, and extra time was allowed after recording for the dye to diffuse into the cell processes (Figure 4E). Morphological reconstructions showed that all examined cells had extensive neurites that resembled cortical neurons (Figure 4F). All mCherry-positive cells in the reprogrammed group showed robust light-induced excitatory post-synaptic currents (EPSCs) (Figure 4G). Surviving neighbors also received considerable excitatory inputs (Figure 4H) qualitatively similar to that of healthy controls (Figure 4J). On the contrary, there were minimal EPSCs in cells in the untreated ischemia group (Figure 4I). The maximal EPSC profile along tangential (Figure 4K) and vertical (Figure 4L) directions averaged across cells for each group revealed no shift in the overall shape of the EPSCs spatial distribution. We then compared the distributions of averaged EPSC amplitudes among the four groups of cells. The reprogrammed cells had significantly larger responses than all the other groups, as shown in the cumulative density curve. To compare the relative connection strength between groups, we transformed the absolute values of EPSC amplitudes to normal distributions using Box-Cox transformation (details see “Methods”). The transformed EPSCs were normally distributed in all four groups. The reprogrammed group had the largest mean of transformed EPSC values, while the untreated ischemia group had the smallest (Figure 4M, inset). The CRACM experiment at 3 weeks post-infection directly demonstrated that functional synaptic inputs from the layer 5 pyramidal neurons of the pre-existing local circuits were formed onto the newly reprogrammed neurons. The relative strength of these projections was stronger than projections onto neurons in the same cortical area without ischemic insult. Surviving endogenous neurons following the ischemic injury in the reprogrammed group had stronger synaptic connections with the circuit than surviving neurons in the untreated ischemia condition.

### Correction of the Circuit Hyperconnectivity and Fast-Spiking Unit Responses 6 Weeks After *in vivo* Direct Reprogramming

**FIGURE 5:**
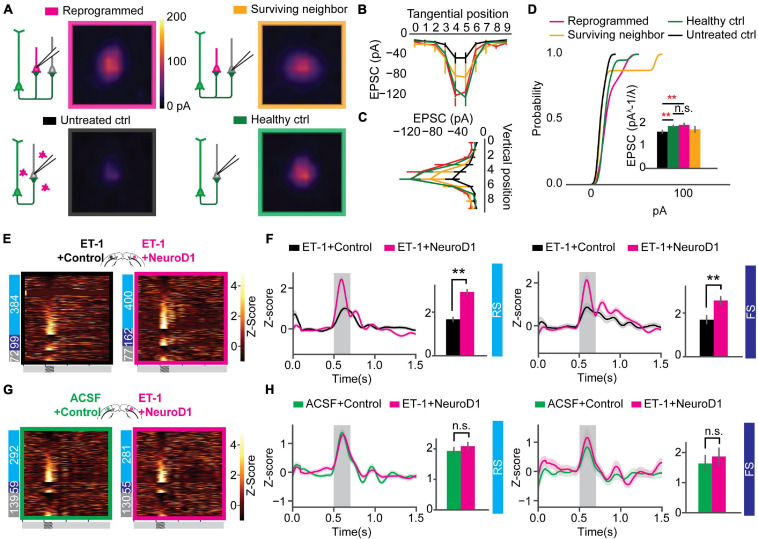
Correction of circuit hyperconnectivity and fast-spiking unit responses at 6 weeks after *in vivo* direct reprogramming. **(A)** Averaged CRACM heat map of reprogrammed cells (*N* = 12 cells from 9 mice), surrounding neighbors (*N* = 8 cells from 9 mice), untreated control (*N* = 16 cells from 6 mice), and healthy control (*N* = 22 cells from 4 mice), measured at 6 weeks after viral infection. **(B)** Averaged maximal EPSC ± SEM by grid position in the tangential direction (parallel to the brain surface). **(C)** Averaged maximal EPSC ± SEM by grid position in the vertical direction (perpendicular to the brain surface). **(D)** The cumulative density curve showing the distribution of pooled EPSCs amplitude in panel **(C)**. The inset bar graph showing Box-Cox-transformed EPSCs amplitude comparison among groups. Reprogrammed vs. Untreated: *p* = 0.00995; Reprogrammed vs. Healthy: *p* = 0.900; Surviving vs. Healthy: *p* = 0.312. One-way ANOVA followed by Tukey *post hoc* tests. **(E)** Firing rate z-scores of all units in heatmaps for “ET-1+Control vs. ET-1+NeuroD1” group. The numbers of RS, FS, and UN units are shown on the left. **(F)** Firing rate z-scores of RS (left) and FS (right) units in the “ET-1+Control vs. ET-1+NeuroD1” group. RS units: N_ET–1__+__Control_ = 384 units, N_ET–1__+__NeuroD1_ = 400 units, *p* = 4.09 × 10^–3^. FS units: N_ET–1__+__Control_ = 99 units, N_ET–1__+__NeuroD1_ = 162 units, *p* = 0.0065. 14 recordings. Mann-Whitney *U*-test with Bonferroni correction. **(G)** Same as **(E)** but for “ACSF+Control vs. ET-1+NeuroD1” group. **(H)** Same as **(F)** but for the “ACSF+Control vs. ET-1+NeuroD1” group. The shaded area represents visual stimulation. Peak z-scores within the visual stimulation window were quantified. RS units: N_ACSF__+__Control_ = 292 units, N_ET–1__+__NeuroD1_ = 281 units, *p* = 0.499. FS units: N_ACSF__+__Control_ = 59 units, N_ET–1__+__NeuroD1_ = 55 units, *p* = 0.540. 9 recordings. Mann-Whitney *U*-test with Bonferroni correction. **p < 0.01 and n.s., not significant. Data are represented as mean ± SEM. See also Supplementary Figures 3, 4.

Circuit connectivity and visual responses improved after the NeuroD1-based gene therapy treatment. However, differences were observed between the reprogrammed and the healthy control groups in both visual responses and circuit connectivity 3 weeks after viral infection. To examine the effect of additional maturation of the reprogrammed neurons on their integration into the local circuits, we further measured circuit connectivity and visual responses 6 weeks after viral injections in mice receiving visual experience in their housing cages. We conducted CRACM on visual cortex slices *ex vivo* to compare the connectivity profile of the newly reprogrammed cells, their surviving neighbors, surviving cells in the untreated ischemia controls, and cells in the healthy controls. The acute brain slices were prepared using the NMDG recovery method (Ting et al., 2018) due to the age of the mice (2.5 months at the time of CRACM, see “Methods” for details). Reprogrammed cells were well connected to local circuits (Figure 5A, top left) as well as their surviving neighbors (Figure 5A, top right). The average connectivity maps of reprogrammed cells and surviving neighbors were comparable to that of the healthy control group (Figure 5A, bottom right) and were more prominent than the untreated ischemia control (Figure 5A, bottom left). The maximal EPSC profile along tangential (Figure 5B) and vertical (Figure 5C) directions averaged across cells for each group revealed no shift in the overall shape of the EPSCs spatial distribution. When comparing the EPSC amplitude, interestingly, the hyper-connectivity to the local circuits in the reprogrammed group was much less prominent compared to the 3 weeks post-infection results. When counting the number of hot spots that had larger than 30 pA EPSC amplitudes, the reprogrammed cells received inputs at less hot spots at 6 weeks post-infection, compared to 3 weeks post-infection (Supplementary Figure 3). The reprogrammed group received significantly stronger projections than the untreated ischemia control group and received equally strong projections than the healthy control group (Figure 5D, inset). In addition, their surviving neighbors received projections that were not significantly different from a healthy control.

Ischemic injury prevalence increases with age (Johnston et al., 2003; Ovbiagele and Nguyen-Huynh, 2011). Therefore, we conducted similar experiments in older adults to test whether the positive effect of reprogramming in treating ischemic injury also applies to old mice over 3 months old. In older adults, the averaged CRACM maps showed the same trend as in young adults that newly converted neurons received robust projections, as well as their surviving neighbors, at a comparable level as in healthy control, and the projections were much more prominent than in untreated ischemia control (Supplementary Figures 4A,B). To account for the overall cortical connectivity change and the concomitant Channelrhodopsin-2 expression level changes over time, we normalized logEPSC values of each group against the healthy control of the same time point. The reprogrammed cells had very prominent hyper-connectivity at 3 weeks after viral infection (Supplementary Figure 4C), the magnitude of which largely decreased 6 weeks post-infection in both young and old adults. The connection strength of the surviving neighbor cells in the reprogrammed mice matched the healthy control at 6 weeks post-infection. In addition, we analyzed input resistance based on membrane potential changes upon current injections for each patched cell. The newly converted cells at 3 weeks after a viral infection had significantly lower input resistance compared to the other groups (Supplementary Figure 4D). This input resistance difference diminished 6 weeks post-infection in both young and old adults, suggesting intrinsic properties of the reprogrammed cells change over time.

Visual responses in the NeuroD1 treated mice also showed continued improvement. In the “ET-1+Control vs. ET-1+NeuroD1” group, not only RS unit firing, but also FS unit firing was significantly higher in the ET-1+NeuroD1 hemispheres than the ET-1+Control hemispheres (Figure 5F). In the “ET-1+NeuroD1 vs. ACSF+Control” group, RS unit firing in the ET-1+NeuroD1 hemispheres was comparable to the ACSF+Control hemispheres (Figure 5H, left). Interestingly, unlike 3 weeks post-infection, FS unit firing was not significantly different from the ACSF+Control hemispheres (Figure 5H, right). This suggests that FS cells have delayed development compared to RS units.

### Orientation Selectivity of the Local Neuronal Population Was Improved Over Time

**FIGURE 6:**
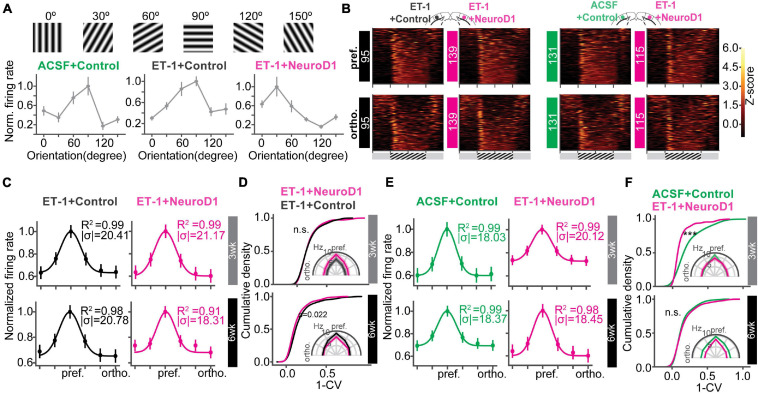
Orientation selectivity of the local neuronal population is improved over time. **(A)** Gratings of 6 orientations used to measure orientation selectivity. Bottom: normalized firing rate of representative units for each group. **(B)** Heatmaps of firing rate z-scores of all units in response to the preferred (pref., top) and the orthogonal (ortho., bottom) orientations for each group. **(C)** The curves showing unit averaged firing rates to 6 orientations normalized and fitted with Gaussian functions of the “ET-1+NeuroD1 vs. ET-1+Control” group at 3 weeks and 6 weeks post-infection. The insets showing the coefficient of determination (R^2^) and sigma of fitted functions. **(D)** Cumulative distributions of 1-CV of units in panel **(C)**. Inset, unit averaged firing rates to 6 orientations. 3 weeks: N_ET–1__+__Control_ = 95 units, N_ET–1__+__NeuroD1_ = 139 units, from 6 mice, *p* = 0.954; 6 weeks: N_ET–1__+__Control_ = 93 units, N_ET–1__+__NeuroD1_ = 142 units, from 4 mice, *p* = 0.022. 2-sample Kolmogorov-Smirnov test. **(E)** Same as **(C)** but for “ET-1+NeuroD1 vs. ACSF+Control” group. **(F)** Same as **(D)** but for “ET-1+NeuroD1 vs. ACSF+Control” group. 3 week: N_ACSF__+__Control_ = 131 units, N_ET–1__+__NeuroD1_ = 115 units, from 4 mice, *p* = 1.659 × 10^–7^; 6 week: N_ACSF__+__Control_ = 183 units, N_ET–1__+__NeuroD1_ = 172 units, from 8 mice, *p* = 0.652, 2 sample Kolmogorov-Smirnov test. ***p < 0.001 and n.s., not significant. Data are represented as mean ± SEM. See also Supplementary Figure 5.

**FIGURE 7:**
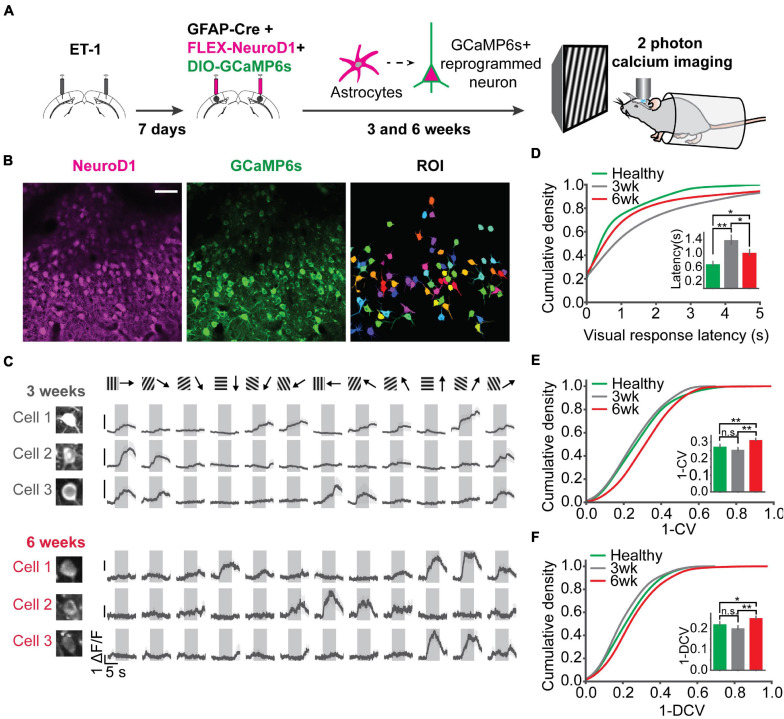
Two-photon calcium imaging reveals response latency decrease and direction selectivity improvement of the reprogrammed neurons over time. **(A)** Injection scheme for two-photon calcium imaging. ET-1 was first injected to induce a focal stroke. 7 days after ET-1 injection, AAV-GFAP::Cre, AAV-FLEX-NeuroD1-mCherry, and AAV-FLEX-GCaMP6s were injected in the same sites. Reprogrammed cell activities in awake mice were imaged by a two-photon microscope at 3 and 6 weeks after viral injection (at the age of 3-3.5 months old). For the healthy control, AAV-syn-jGCaMP7s was injected into mouse V1, and cell activities were imaged at 3-4 weeks post-injection (at the age of 2.5 months old). **(B)** NeuroD1 and GCaMP6s signals were well overlapped. Well isolated ROIs were chosen for further analysis. Scale bar represents 50 μm. **(C)** Trial-averaged traces of representative cells showed selective responses to 12 directions. Shaded areas represent visual stimulation. **(D)** The cumulative density of visually evoked response latencies of cells at 3 and 6 weeks after viral injection, compared with cells in healthy control mice. Quantification of latencies shown in the inset. N_3 week_ = 187 ROIs from 3 mice, N_3 week_ = 181 ROIs from 5 mice, N_6 week_ = 375 ROIs from 5 mice. 3 week vs. 6 week: *p* = 0.023; 3 week vs. Healthy: *p* = 0.001; 6 week vs. Healthy: *p* = 0.045, One-way ANOVA followed by Tukey *post hoc* tests. **(E)** Cumulative density curves of 1-CV of the reprogrammed cells at 3 and 6 weeks after viral injection. Quantification of 1-CV shown in the inset. 3 week vs. 6 week: *p* = 0.001; 3 week vs. Healthy: *p* = 0.392; 6 week vs. Healthy: *p* = 0.005, One-way ANOVA followed by Tukey *post hoc* tests. **(F)** Same as **(E)** but for 1-DCV. Quantification of 1-DCV shown in the inset. 3 vs. 6 week: *p* = 0.001; 3 week vs. Healthy: *p* = 0.341; 6 week vs. Healthy: *p* = 0.036, One-way ANOVA followed by Tukey *post hoc* tests. *p < 0.05, **p < 0.01, and n.s., not significant. Data are represented as mean ± SEM.

Following postnatal development and visual experience, most V1 neurons acquire preference to a specific orientation (Li et al., 2008; Nauhaus et al., 2008; Ko et al., 2011). To further assess functional recovery following reprogramming, we examined neuronal unit activity in response to sinusoidal gratings of 6 orientations (Figure 6A). Representative units and population firing rate z-scores to different orientations revealed selective responses of most units in each group (Figures 6A,B). To quantify population selectivity to orientations, we averaged firing rate across units to 6 orientations and fitted Gaussian functions for each group to estimate “population tuning curves.” 3 weeks post-infection, in the “ET-1+NeuroD1 vs. ET-1+Control” group, tuning curve width (variance (σ) of the fitted function) was 20.957 degrees for the ET-1+NeuroD1 hemisphere, only slightly sharper than 21.322 degrees for the ET-1+Control hemisphere (Figure 6C, top). At the same time, in the “ET-1+NeuroD1 vs. ACSF+Control” group, the ET-1+NeuroD1 hemisphere tuning curve width was 20.387 degrees, broader than 17.995 degrees of the ACSF+Control hemisphere (Figure 6E, top). However, 6 weeks post-infection, in the “ET-1+NeuroD1 vs. ET-1+Control” group, the tuning curve width was 17.504 degrees for the ET-1+NeuroD1 hemisphere, sharper than 22.880 degrees for the ET-1+Control hemisphere (Figure 6C, bottom). In the “ET-1+NeuroD1 vs. ACSF+Control” group, the ET-1+NeuroD1 hemisphere tuning curve width was 18.228 degrees, comparable to 17.885 degrees for the ACSF+Control group (Figure 6E, bottom). To quantitatively compare orientation selectivity distributions between groups, we calculated the orientation selectivity index (one minus the circular variance of firing rates to 6 orientations, 1-CV) for each unit (Figures 6D,F, see section “Method” for details). In the “ET-1+NeuroD1 vs. ET-1+Control” group, the cumulative distribution of 1-CV of the ET-1+NeuroD1 hemisphere showed no difference compared to the ET-1+Control hemisphere at 3 weeks, and marginal difference at 6 weeks post-infection. While in the “ET-1+NeuroD1 vs. ACSF+Control” group, the cumulative distribution of 1-CV of the ET-1+NeuroD1 hemisphere was left-shifted compared to the ACSF+Control hemisphere at 3 weeks but was not different at 6 weeks post-infection. These results revealed that the orientation tuning of the cortical population was not completely recovered 3 weeks post-reprogramming but was comparable to the sham condition 6 weeks post-reprogramming. However, the orientation selectivity of the NeuroD1 group could be the result of either improvement in the reprogrammed neurons or pre-existing neurons, or both. To answer this question, we used 2-photon calcium imaging to look at the visual responses of the reprogrammed cells to drifting gratings of 12 directions, and compared to the healthy controls. The specific expression of GCaMP6s in reprogrammed neurons was achieved by injecting AAV-CAG::FLEX-GCaMP6s together with AAV-GFAP::Cre and AAV-FLEX-NeuroD1-mCherry (Figure 7A). Most reprogrammed cells expressed GCaMP6s, and could be identified as well-separated regions of interest (ROIs, Figure 7B). The reprogrammed cells showed responses to visual stimuli at both three and 6 weeks after viral injection, while the response latency and selectivity to directions were further improved 6 weeks post-infection (Figure 7C). The cumulative density curve of visually evoked response latencies of the reprogrammed was left-shifted 6 weeks post-infection compared to the distribution 3 weeks post-infection (Figure 7D). The response latencies of the reprogrammed cells were longer than the healthy control cells at 3 weeks post-infection, and the difference was reduced at 6 weeks post-infection. The percentage of neurons whose response latencies were less than 3 s was up to 89.6% 6 weeks post-infection, which suggested that most cells showed typical neuronal visual response latency. Furthermore, the distributions of orientation and direction selectivity indices (1-CV, 1-DCV) were both right-shifted 6 weeks post-infection compared to the distribution 3 weeks post-infection (Figures 7E,F). The orientation and direction selectivity of the reprogrammed cells were lower compared to the healthy control at 3 weeks post-infection. However, the selectivity indices of the reprogrammed cells were higher than the healthy control at 6 weeks post-infection. The percentage of cells that had orientation selectivity index (1-CV) larger than 0.4, indicative of a mature selective neuron, increased from 16.02% at 3 weeks post-infection (*n* = 29 cells, n_total_ = 181 cells) to 28% at 6 weeks post-infection (*n* = 105 cells, n_total_ = 375 cells), and the percentage of cells that had direction selectivity index (1-DCV) larger than 0.4 increased from 8.83% at 3 weeks post-infection (*n* = 16 cells, n_total_ = 181 cells) to 14.93% at 6 weeks post-infection (*n* = 56 cells, n_total_ = 375 cells). In addition to 2-photon imaging, we used optotagging to examine orientation selectivity of the reprogrammed neurons by injecting AAV-DIO-ChR2-eYFP together with AAV-GFAP::Cre and AAV-FLEX-NeuroD1-mCherry (Supplementary Figure 5A). We identified 22 cells that reliably responded both to optogenetic and visual stimulation and found most of them were selective to orientations (Supplementary Figures 5A–H). Both *in vivo* 2-photon calcium imaging at two developmental times and optotagging results suggest that the reprogrammed cells acquire orientation- and direction-selective responses over time and indicate their functional integration into the local visual cortical circuits.

## Discussion

### Characterization of Neuronal Circuit Functions Is a Critical Assessment of the Therapy

We demonstrated that NeuroD1-mediated *in vivo* direct reprogramming of astrocytes into neurons promoted their neural circuit integration and led to the visual functional recovery after ischemic injury. Our work bridged the knowledge gap between individual cellular response recovery and animal behavioral recovery, where we characterized the functional synapses formed from specific projections and assessed neuronal response to stimuli in awake mice, which are critical functional characterization at the intermediate neural circuit level. The mouse primary visual cortex is a unique model system providing an opportunity to quantify projection specific functional connectivity and the direct visual responsiveness of the reprogrammed cells. Furthermore, the ability to record responses to different visual features such as orientation and direction provides a unique ability to quantify how well the cells mature and whether the synapses they receive are functional.

### Visual Response Recovers and Selectivity to Orientations Sharpens Following the Therapy

In our model system, the visual responses were drastically reduced following ischemic injury, yet they recovered following the NeuroD1 delivery. The putative excitatory neurons started to regain their visual responses 3 weeks after reprogramming, while the putative inhibitory neurons progressively integrated circuit inputs and refined their activity over a longer period of time. This delayed recovery of inhibition after reprogramming is similar to the absence of matured inhibition at an early age during postnatal V1 development (Minlebaev et al., 2011; Shen and Colonnese, 2016). Furthermore, these visual responses became more specific with time, based on our two-photon calcium imaging and extracellular recording results. The NeuroD1 converted cells gradually developed to be selective to the orientations and directions of visual stimuli, which is a typical feature of the mature visual cortical neurons. Interestingly, the reprogrammed cells at 6 weeks post-infection demonstrated higher selectivity compared to the healthy controls, which could be potentially explained by the more functionally developed synaptic inputs received by the reprogrammed cells compared to the healthy controls.

### Local Functional Circuits Undergo Refinement of Synaptic Inputs

At the same time, using the functional circuit mapping technique, we gained more insights into synaptic input integration during the recovery process. We observed initial functional synapse formation and even hyper-connectivity in newly reprogrammed neurons 3 weeks after viral injection. Initially, the reprogrammed neurons received almost twice as many synapses compared to the healthy control cells. The excessive synapse numbers decreased 6 weeks after reprogramming. These functional synapses represented appropriate and not aberrant synapses, characteristic for the primary visual cortex circuitry. As the reprogrammed cells assume neuronal fate and undergo synaptogenesis, more inputs drive the cell activity, leading to the pruning of excess synapses, activity refinement, and cell maturation. This process also coincides with the maturation of inhibition seen in our *in vivo* work. Strikingly, surviving neurons also regained synaptic connections, suggesting that removing the glial scar might help rewire the damaged brain area (Zhang et al., 2018). This finding suggests that structural and functional synaptic plasticity may be present in the newly reprogrammed neurons, resembling neonatal neuronal development processes (Chechik et al., 1999).

### Visual Experience Might Play a Role in Activity Refinement Following the Therapy

Visual experience in the housing cages may have led to the pruning of excess synapses, activity refinement, and cell maturation. Slower development of inhibitory responses is consistent with the delayed maturation of inhibition following visual experience in the normal developing cortex. Improved orientation selectivity of the reprogrammed neurons with additional time and visual experience may result from their integration into the local circuits and maturation of the local inhibition (Liu et al., 2011).

In our work, the demonstration of visual response and selectivity recovery provides a novel cellular and circuit characterization consistent with the previous work (Chen et al., 2020). CRACM experiments provided the quantification of the functional synaptic connectivity recovery extending the prior report of non-specific spontaneous synaptic currents developing in reprogrammed cells (Guo et al., 2014; Chen et al., 2020). We discovered, for the first time to our knowledge, that the functional maturation of the reprogrammed neurons shares similarities with the typical postnatal cortical circuit development. This finding suggests the importance of experience in the development of the reprogrammed cells and functional brain recovery after injury.

### Conversion Efficiency and Functional Recovery Are Similar to Other Therapies

Compared to other studies, the functional recovery achieved by NeuroD1-mediated astrocyte-to-neuron conversion *in vivo* was similarly efficient. The reprogrammed neurons in the visual cortex acquired the cortical layer structure, similarly to Ngn2- and Nurr1-mediated reprogramming (Mattugini et al., 2019). The local functional circuit and visual response recovery were also similar to embryonic neuronal transplantation results (Falkner et al., 2016). However, other methods such as cell transplantation may have side effects, such as immune response, which limit their therapeutic potential. Direct *in vivo* conversion of astrocytes into neurons removes the possibility of graft rejection and provides a viable solution for this problem.

Our findings suggest that the NeuroD1-based *in vivo* direct reprogramming technology may be a promising gene therapy treatment of brain injury by replenishing the lost neurons and successfully integrating them into the existing neural circuit.

## Data Availability Statement

The raw data supporting the conclusions of this article will be made available by the authors, without undue reservation.

## Ethics Statement

The animal study was reviewed and approved by the Purdue University Animal Care and Use Committee.

## Author Contributions

QW, YT, MG, GC, and AC designed the experiments. ZP produced the viruses. YT, QW, YC, AR, ZX, TW, and WL performed the IHC experiments. YT and SK performed the extracellular recordings. QW performed the CRACM experiments. MG performed 2 photon calcium imaging. YT, QW, and ER performed the injections. YT, QW, MG, ZX, TW, and WL analyzed the data and drafted the manuscript. YT, QW, MG, GC, and AC edited and revised the manuscript. All authors contributed to the article and approved the submitted version.

## Conflict of Interest

GC was a co-founder of the NeuExcell Therapeutics Inc. The remaining authors declare that the research was conducted in the absence of any commercial or financial relationships that could be construed as a potential conflict of interest.

## Publisher’s Note

All claims expressed in this article are solely those of the authors and do not necessarily represent those of their affiliated organizations, or those of the publisher, the editors and the reviewers. Any product that may be evaluated in this article, or claim that may be made by its manufacturer, is not guaranteed or endorsed by the publisher.
