# Supplementary material for: Restoration of Visual Function and Cortical Connectivity After Ischemic Injury Through NeuroD1-Mediated Gene Therapy
Source: Front Cell Dev Biol. 2021 Aug 18;9:720078. doi: 10.3389/fcell.2021.720078 (PMC8416524; doi:10.3389/fcell.2021.720078)
Supplement: Supplementary file 1 [file Data_Sheet_1.docx]

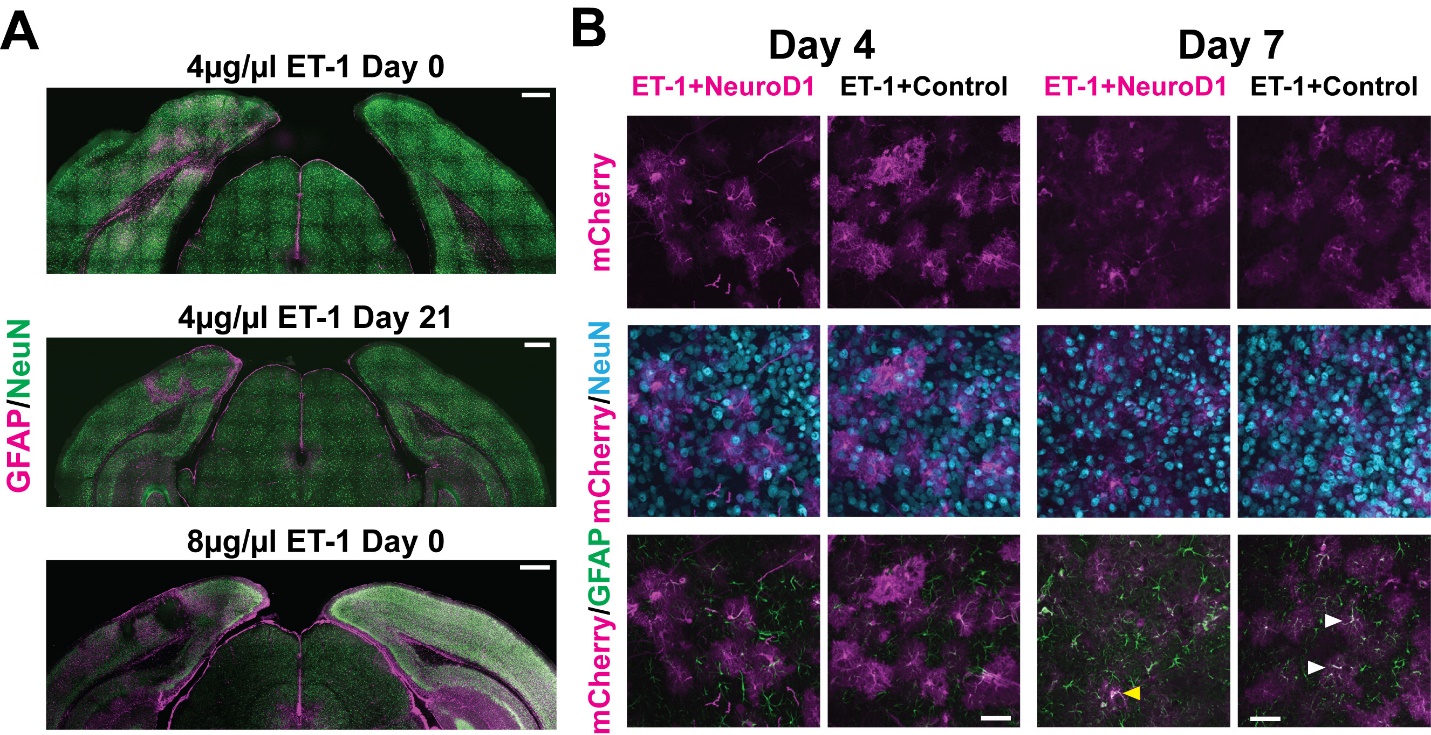


**Supplementary Figure 1. Gliosis and neuronal loss in ET-1 injected visual cortex and cell conversion following *in vivo* direct reprogramming in the primary visual cortex.**

(A) Gliosis (GFAP, magenta) and neuronal loss (NeuN, green) at 9 days after 4ug/ul ET-1 injection (top left hemisphere). Persisted gliosis after 4 weeks (middle left hemisphere). Larger tissue damage induced by 8ug/ul ET-1 (bottom left hemisphere). ACSF injected control (right hemispheres). Scale: 500µm.

(B) mCherry positive cells co-stained­ with NeuN in the reprogrammed group and GFAP in control at 4 and 7 days after viral injection. Arrows pointing to double positive cells. Scale: 50µm.


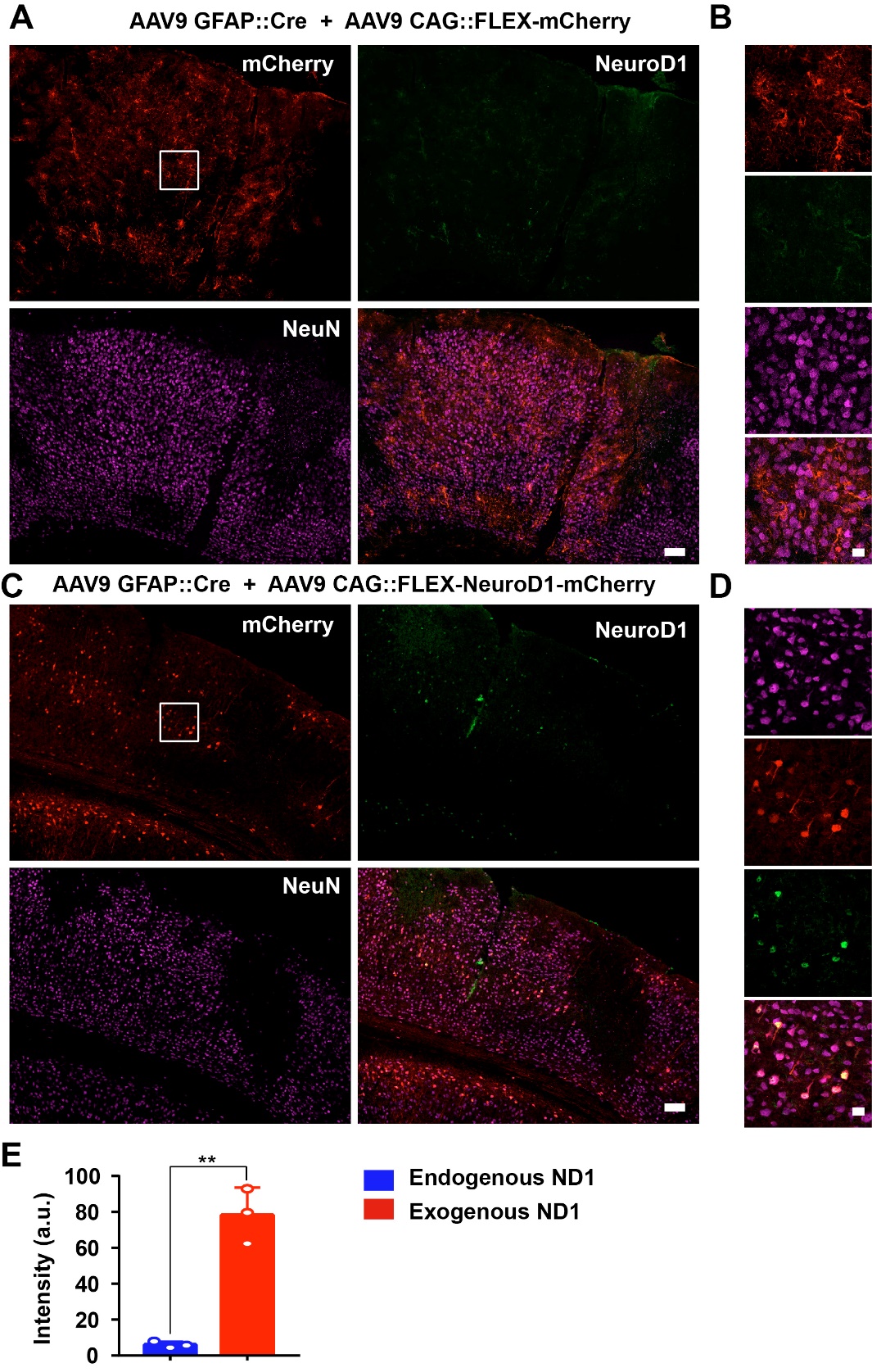


**Supplementary Figure 2. Comparison of endogenous NeuroD1 expression level versus AAV overexpressed NeuroD1 after astrocyte-to-neuron conversion.**

(A-B) Representative images illustrating that in the control mCherry group, the endogenous neurons (labeled by NeuN) rarely showed detectable level of NeuroD1 expression in the mouse cortex. Scale bar for panel A, 100 mm. Panel B shows the enlarged box area in panel A. Scale bar for panel B, 20 mm.

(C-D) Representative images showing that in the NeuroD1 group, after astrocyte-to-neuron conversion (30 days post AAV NeuroD1 infection), NeuroD1 immunostaining (green) detected clear NeuroD1 signal in the NeuroD1-mCherry-converted neurons (red). Scale bar for panel C, 100 mm. Panel D shows the enlarged box area in panel C. Scale bar for panel D, 20 mm.

(E) Quantitative analysis showing that the exogenous NeuroD1 expression level in NeuroD1-mCherry-converted neurons was significantly higher than the endogenous NeuroD1 expression level. **P < 0.01, Student’s t-test (n = 3 animals for each group).


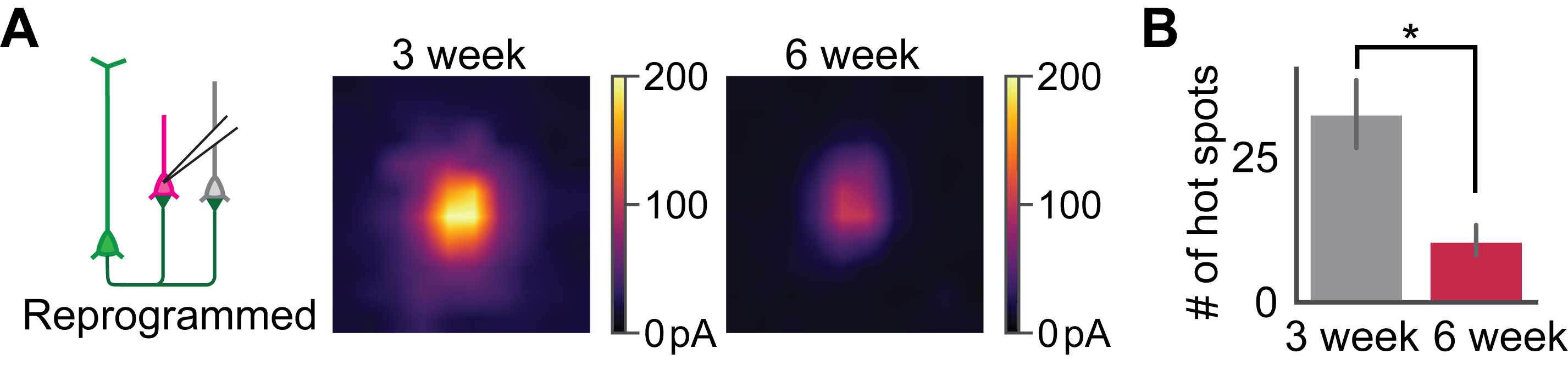


**Supplementary Figure 3. The reprogrammed cells received less synaptic inputs at 6 weeks compared to 3 weeks post-injection.**

(A) Averaged CRACM maps of reprogrammed cells at 3 weeks and 6 weeks post-injection.

(B) Quantification of the numbers of CRACM heatmap hot spots that had larger than 30pA EPSC amplitude. N_3week_=21 cells from 6 mice, N_3week_=12 cells from 9 mice. Mann-Whitney U test.

*-p<0.05. Data are represented as mean ± SEM.


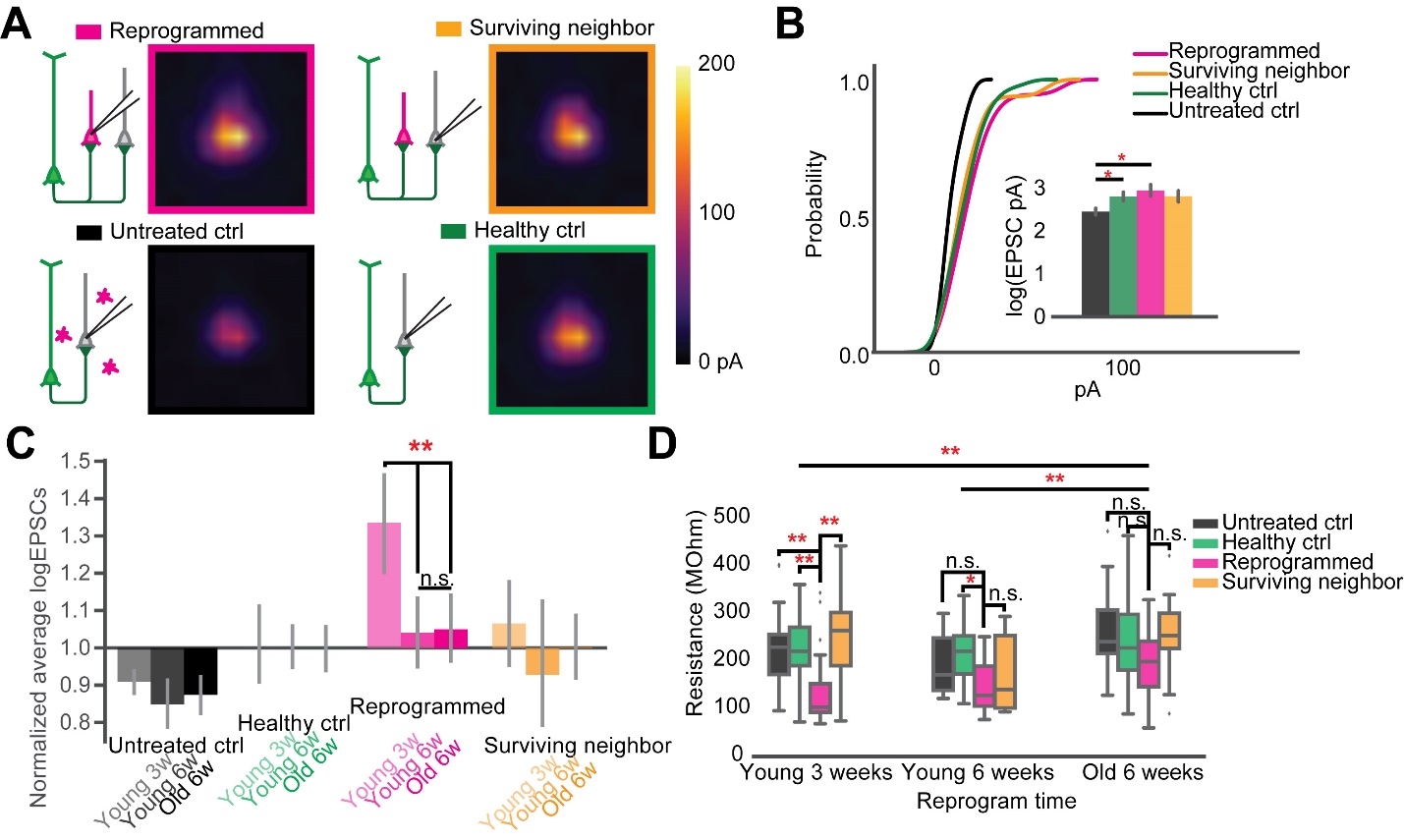


**Supplementary Figure 4. Circuit repair restoration with *in vivo* direct reprogramming after ischemic injury were consistent in older adult mice.**

(A) Averaged CRACM heat map of reprogrammed cells (N=18 cells from 9 mice), surrounding neighbors (N=16 cells from the same cohort of 9 mice), untreated control (N=28 cells from 9 mice), and healthy control (N=34 cells from 8 mice), measured at 6 weeks after viral infection in animals older than 3 months.

(B) The cumulative density curve showing the distribution of pooled EPSCs amplitude in (A). The inset bar graph showing comparison of the log-transformed EPSCs amplitude among all groups. Reprogrammed vs. Untreated: p=0.0135; Untreated vs. Healthy: p=0.048. One-way ANOVA followed by Tukey post-hoc tests.

(C) logEPSC at each time point normalized against the sham control. Two-way ANOVA, Groups: F(3)=15.941, p=1.984x10^-9^; Timepoints: F(2)=5.467, p=4.81x10^-3^; interaction: F(6)=2.532, p=0.064. Tukey's HSD test, untreated group: Young 3w vs. Old 6w p=0.603; Young 3w vs. Young 6w p=0.680; Young 6w vs. Old 6w p=0.900. Reprogrammed: Young 3w vs. Young 6w p=0.00433; Young 3w vs. Old 6w p=0.00157, Young 6w vs. Old 6w p=0.900. Surviving: Young 3w vs. Young 6w p=0.375; Young 3w vs. Old 6w p=0.711, Young 6w vs. Old 6w p=0.732. Tukey’s HSD test.

(D) The comparison of input resistance at each time point. Two-way ANOVA, Groups: F(3)=9.41, p = 7x10^-6^; Timepoints: F(2)=7.884, p=0.000493; interaction: F(6)=0.726, p=0.629. Tukey's HSD test, among Groups at Young 3 weeks: Reprogram vs. Surviving p=0.00115; Reprogram vs. Untreated p=0.00562; Reprogram vs. Healthy p=0.00506; Young 6 weeks: Reprogram vs. Healthy: p=0.0187; p>0.05 for all other pairs of comparison at Young 6 weeks and Old 6 weeks. Old 6 weeks vs. Young 6 weeks: p=0.00771; Old 6 weeks vs. Young 3 weeks: p=0.00113; Young 3 weeks vs. Young 6 weeks p=0.685. Tukey post-hoc tests.

*-p<0.05, **-p<0.01, ***-p<0.001, n.s.-not significant. Data are represented as mean ± SEM.


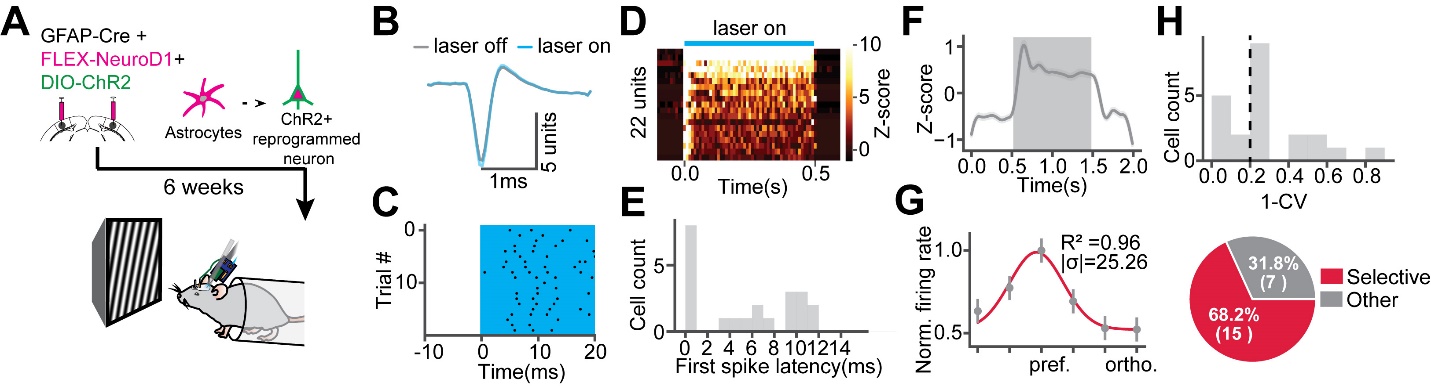


**Supplementary Figure 5. Optogenetically tagged reprogrammed cells are visually responsive and orientation selective.**

(A) AAV-DIO-ChR2 was injected together with AAV-GFAP::Cre and AAV-FLEX-NeuroD1 to express ChR2 in the reprogrammed cells. 6 weeks after reprogramming, visual cortical activities were measured by extracellular recordings with optogenetic stimulation.

(B) Mean template waveforms of a representative unit during laser off and laser on.

(C) Raster plot of the cell in (B) during optogenetic stimulation. The cyan shade indicates optogenetic stimulation.

(D) Firing rate z-scores of units in response to optogenetic stimulation. 22 units that have trial-median first spike latencies shorter than 15ms and mean firing rate z-scores within the optogenetic time window are defined as optotagged units.

(E) The distribution of first spike latencies of units in (D).

(F) Average firing rate z-scores of optotagged units in response to visual stimulation. The shaded area represents visual stimulus time window.

(G) Average firing rates of optotagged units in response to 6 orientation are normalized and fitted with a Gaussian function. The inset showing the coefficient of determination (R^2^) and sigma of fitted functions.

Data are represented as mean ± SEM.
